# Retrospective Editorial

**DOI:** 10.1098/rsbl.2024.0581

**Published:** 2024-12-04

**Authors:** David Beerling

**Affiliations:** ^1^ Editor-in-Chief

*Biology Letters* (https://royalsocietypublishing.org/journal/rsbl) is a Royal Society journal focused on the rapid publication of short high-quality research articles, reviews and opinion pieces across the biological sciences. After almost 7 years as Editor-in-Chief (EiC), I will be completing my second and final term at end of this year. It has been a huge pleasure to work with the outstanding editorial team and staff over this time to lead and develop the journal. The journal has continued to evolve and address the many challenges we face in a constantly changing publishing landscape to provide the best service to our authors and readers. In this editorial, I highlight some of these challenges and the developments we have undertaken over my time as EiC. These include our move to open access, the growing number of competitor journals in our subject fields, the appointment of our Preprint Editor, as well as the establishment of our Early Career Researcher (ECR) Prize. We also, of course, dealt with surviving the challenge of the COVID-19 pandemic [1].

In terms of open access, I would like to highlight that *Biology Letters* is a Plan S compliant Transformative Journal (https://royalsociety.org/journals/publishing-metrics/). Moreover, we remain committed to becoming completely open access in the near future. At the time of writing, more than half of our content is open access, and we are confident this will continue to increase over time.

The launch of similar biological journals in recent years has provided researchers with more outlets for their work. Recognizing this, we listened to the needs of authors and at the start of 2024 increased our word limit from 2500 to 3500 words, with the aim of keeping article lengths more or less in-line with our competitors, while remaining a home for short research articles. I am pleased to report that our submissions are rising strongly through 2024, a trend reflecting both the attractiveness of the journal format and its reputation. It is also testament to the hard work of the editorial team advocating the journal on social media platforms and in-person at conferences.

We are also witnessing the rise of artificial intelligence (AI) and, in a previous editorial [2], I highlighted the steps the journal, in common with other Royal Society journals, is taking to address this issue. Currently, in *Biology Letters*, authors must declare whether they have used AI in their manuscript. Declaring the use of these technologies supports transparency and trust, and the editorial team will continue to review our policy as AI continues to evolve.

Among the many developments over the past few years has been the rise in popularity of preprint repositories. At *Biology Letters,* we embraced this development with the appointment of our Preprint Editor, Catherine Talbot, who is actively recruiting more people to join her team and expand the subject area coverage [2]. Please do contact the editorial office if you would like to know more about this. We also encourage researchers to deposit pre-publication versions of articles in appropriate preprint repositories such as bioRxiv, paleorXiv and EcoEvoRxiv.

I was delighted to have introduced our ECR Prize last year [3]. Our inaugural ECR winner, Joe Wynn at the Institut für Vogelforschung, Germany, was announced at the end of October 2023. Joe won the award for his highly original contribution to our understanding of inherited orientation in song birds returning from migration [4]. Many congratulations to Joe and the two runners-up, Ana Valenzuela-Toro (University of California, Santa Cruz) and Antoine Guiguet (Pennsylvania State University) for their work on how feeding morphology and body size shape resource partitioning in an eared seal community, and extreme acidity in a cynipid gall [5,6], respectively. The competition to find our 2024 prize winner is underway and we look forward to announcing the 2025 competition in due course.

As always, I would like to thank our talented and dedicated authors, readers and reviewers for their hard work over the past year. I remain continually astonished at the scientific diversity and creativity of our authors, which are also often featured in the press. Recent examples include artificial light at night affecting spider brains (https://theconversation.com/city-light-pollution-is-shrinking-spiders-brains-238086) and dogs remembering the names of objects (https://www.theguardian.com/science/article/2024/sep/04/dogs-remember-names-toys-years-study-pets-memory).

The success of the journal depends on their efforts, together with those of our Editorial Board and publishing team. It is a pleasure to thank our excellent editorial and production office of the Royal Society who provide outstanding support to the EiC and the journal as whole, including our 90+ strong international (https://royalsocietypublishing.org/rsbl/editorial-board), composed of expert Board Members, and Handling, Reviews and Preprint Editors.

Next year is a special one for *Biology Letters*—our 20th Anniversary. This is an important milestone for the journal and the editorial team are working hard on preparations for the celebration. Brian Charlesworth FRS, the first EiC of *Biology Letters*, provided an excellent account of our origins story in his own editorial article [7]. Back in 2005, the journal’s emphasis was on rapid turnaround times, an impressive international board and being a natural home for short-format ecological, behavioural and evolutionary biology papers. All of this still holds today, but we now encompass a broader range of subject areas including global change biology, palaeontology and many other topics. Central to this is our successful series of Special features (https://royalsocietypublishing.org/rsbl/special-features), which are collections of articles around a broad theme of topical importance proposed by our authors. A recent feature was a well-received collection of articles on insect decline (https://royalsocietypublishing.org/topic/special-collections/insect-decline). In the pipeline, we have an exciting new Special feature on dinosaur science that is being guest-edited by Paul Barrett and Susannah Maidment from the Natural History Museum. As always, we welcome suggestions for future Special features, as well as your review and opinion piece ideas.

Finally, I would like to extend a warm welcome to Professor Louise Heathwaite FRS from Lancaster University, who from 1 January 2025, will take over as our new EiC. I wish her well for her upcoming tenure.
